# Supplementary material for: Insulin signaling regulates longevity through protein phosphorylation in Caenorhabditis elegans
Source: Nat Commun. 2021 Jul 27;12:4568. doi: 10.1038/s41467-021-24816-z (PMC8316574; doi:10.1038/s41467-021-24816-z)
Supplement: Supplementary file 1 — Supplementary Information [file 41467_2021_24816_MOESM1_ESM.pdf]

Insulin Signaling Regulates Longevity Through Protein Phosphorylation in *Caenorhabditis elegans*

Wen-Jun Li[1,2,10], Chen-Wei Wang[3,4,10], Li Tao[2,6,10], Yong-Hong Yan[2,10], Mei-Jun Zhang[2,7], Ze-Xian Liu[3,8], Yu-Xin Li[2,9], Han-Qing Zhao[2], Xue-Mei Li[1,2], Xian-Dong He[2], Yu Xue[3,4,*] & Meng-Qiu Dong[2,5,*]

Supplementary Information:

Supplementary Discussion

Supplementary Figure 1-8

Supplementary Table 1-2

Supplementary Discussion

Extensive phosphorylation regulation orchestrated by IIS kinases controls lifespan regulation

Previous studies investigating how reduced IIS extends lifespan have focused on transcriptional regulation by FOXO. While it is clear that DAF-16/FOXO activation is required for the long lifespan of the *daf-2* mutant, it is an open question whether DAF-16 activation is sufficient for *daf-2* longevity. Here, we looked into this issue by analyzing the phosphorylation changes against previously documented protein and mRNA abundance changes[1, 2]. For EIF-2α S49 and CDK-1 pT179, two of the three phosphorylation sites that have been characterized in-depth in this study, their parent proteins and the encoding mRNAs show no obvious change in the *daf-2* mutant; for AKT-1 pS492, there is an abundance change of the parent protein but not the encoding mRNA (Fig. 7). Notably, phosphorylation changes at these sites all affect lifespan. In particular, increased EIF-2α pS49 contributes significantly to *daf-2* longevity, and this regulation occurs specifically at the PTM level, not at the mRNA or protein level. Besides, hyper-phosphorylation of EIF-2α at S49 persisted in the *daf-16; daf-2* double mutant (Supplementary Fig. 6f), suggesting a DAF-16-independent change.

Likewise, we found 158 DAF-16-independent and 100 DAF-16-dependent phosphorylation changes (Supplementary Data 3). KEGG-based enrichment analysis demonstrated that there were different regulatory modes between the two groups (Supplementary Fig. 8a). The phosphorylation levels of proteins involved in various pathways such as FOXO signaling, longevity regulating, and glycerophospholipid metabolism pathways were markedly regulated in a DAF-16-dependent manner, whereas those in RNA degradation were independent of DAF-16. Phosphorylation changes on proteins of the mTOR signaling pathway or glycerolipid metabolism were regulated in both DAF-16-dependent and DAF-16-independent fashion, suggesting complex crosstalk between IIS and other longevity pathways. Gene Ontology (GO) analysis showed that GO terms related to cell cycle and protein synthesis were significantly enriched from the DAF-16-independent phosphorylation (either the entire group or the hypo-phosphorylated group, Supplementary Fig. 8b). Importantly, our present

study as well as previous evidences confirmed that retarding the cell cycle or mRNA translation results in lifespan extension, indicating DAF-16-independent phosphorylation events as IIS-related lifespan regulation mechanisms. Taken together, our phosphoproteomics and functional studies suggested that DAF-16 mediated transcriptional regulation alone may be insufficient for *daf-2* longevity.

Functional analysis results from this study indicated that the phosphoproteins from the reproductive system do affect lifespan. Indeed, there are often negative correlations between the reproduction- and lifespan-related phenotypes upon mutating these phosphoproteins. Consider that adult-specific knockdown of genes including *cdk-1*, *chk-2*, *hoe-1*, *hsr-9*, and *htp-3* prevents cell cycle progression and results in lifespan extension, whereas early death phenotypes result from knockdown of the germline hyperproliferation suppressor *gld-1* or the oogenesis-restricting *puf-3*. The data endorsed the antagonistic pleiotropy theory, which proposes that ageing is an adaptation to natural selection of pleiotropic genes that benefit fitness in early life, but are detrimental later[3]. Assayed individually, these hypo-phosphorylated proteins of the reproductive system exhibit only small effects. However, we speculated that they may exert larger effects when combined. Notably, we did not detect clear patterns for mRNA- or protein-level changes for these phosphoproteins in the *daf-2* mutant (Fig. 7).

Regarding LiRKs predicted to regulate differentially phosphorylated in the *daf-2* mutant, a survey of their lifespan phenotypes reported in the literature and our target analysis of the CK2 kinase KIN-3/KIN-10 suggested that, for the most part, their reduced expressions or activities contribute positively to *daf-2* longevity. We also adopted the Kinase-Substrate Enrichment Analysis (KSEA)[4] to estimate kinase activities in the *daf-2* mutant (Supplementary Fig. 8c). A total of 50 kinases were significantly ($p < 0.05$) enriched and all showed a predicted downregulation of kinase activity in *daf-2* worms. However, only 9 of the 50 KSEA-enriched kinases are known lifespan regulators, a proportion lower than that from our analysis (10/27). KIN-3 was also enriched by KSEA but not among the top 27. Our analysis is thus efficient to infer kinases that are involved in *daf-2* longevity. As no clear pattern of changes was evident for these kinases in the *daf-2* mutant at the mRNA or protein-abundance levels (Fig. 7), our study underscored the utility of phosphoprotein-based surveys for

elucidating the impacts of insulin signaling on longevity specifically and for deepening biological understanding generally.

EIF-2α phosphorylation links IIS-modulated amino acid metabolism to translation and longevity

The *daf-2(lf)* mutation led to reduced levels of ribosomal proteins and polyribosome associated RNAs, suggesting a repression of global mRNA translation. Previous studies reported that that *tts-1*, a long noncoding RNA, was required for the reduction of ribosome-associated RNA in *daf-2* worms[5]. We found here that GCN-2/EIF-2α phosphorylation signaling bridges translation and longevity in *daf-2* worms (Fig. 4f and 7). In eukaryotes, eIF2α phosphorylation converts eIF2-GDP into a competitive inhibitor of eIF2B, thus dominantly reducing pre-initiation complex assembly and general translation initiation[6]. Paradoxically, EIF-2α phosphorylation stimulates translation of *atf-5*, a TF homologous to yeast GCN4 and mammalian ATF4[7]; phospho-eIF2α-induced ATF4 expression is required for tumor cell survival and embryonic stem cell proliferation[8, 9]. However, knocking out *atf-5* had no effect on the lifespan of WT or *daf-2* worms (Supplementary Data 4), indicating that the pro-longevity effect of EIF-2α phosphorylation mainly results from retarding general translation, rather than from promotion of ATF-5 translation. It remained to be clarified whether phospho-EIF-2α targets specific mRNA translation to promote lifespan. It was notable that EIF-2α S49A did not fully restore the peak heights of ribosomal fractions in *daf-2* (Fig. 4c), suggesting additional forms of regulation, for example *tts-1* associated ribosome reduction, was apparently involved.

The eIF2α kinases GCN2 and PERK are activated by amino acid depletion and ER stress, respectively. Our data suggested that GCN-2, not PEK-1, mediates the hyper-phosphorylation of EIF-2α S49 and promotes *daf-2* longevity. This was consistent with previous reports that *pek-1* null status neither abrogated ER stress resistance nor shortened the long lifespan of *daf-2* worms[10]. The next question is how the *daf-2* mutation activates GCN-2: several lines of evidence suggest that GCN-2 activity might respond to the IIS-modulated amino acid metabolism. First, uncharged tRNAs or ribosomal stalling directly stimulate GCN2 upon amino acid starvation[11, 12]. Also, knockdown of worm

tRNA synthetases induces EIF-2α S49 phosphorylation through GCN-2[7]. Additionally, reduction of *daf-2* activity lowers the abundance of tRNA synthetases and amino acid pools in young adult worms[13, 14, 15]. It therefore seemed plausible that a shortage of tRNA synthetases and amino acids could lead to loss of tRNA charging and/or ribosome pausing, therefore stimulating GCN-2, a scenario that would support a role for GCN-2/EIF-2α signaling in altering amino acid metabolism to ameliorate translation and longevity in *daf-2* animals.

Germline phosphoproteins mediate the effects of IIS on reproduction and lifespan regulation

daf-2 and *daf-16* mainly function in neurons, the hypodermis, and intestine to regulate lifespan[16, 17, 18]. On the one hand, ablation of germline precursor cells further extends the lifespan of *daf-2* worms, suggesting that IIS acts in parallel with germline signaling to regulate lifespan[19]. On the other hand, IIS is required for germline cell cycle progression to ensure robust germline proliferation[20]. Recent data are starting to indicate a link (probably orchestrated by IIS-regulated SUMOylation) between lifespan regulation by germline signaling and IIS[21].

Here, we found that IIS actively induces phosphorylation on hundreds of germline proteins involved in the cell cycle, apoptosis, translation, etc., indicating pronounced functional impacts from phospho-modulation of germline proteins. As an example, we showed that hypo-phosphorylated CDK-1 T179, which inactivates CDK-1 and thereby delays the cell cycle, potentially contributes to *daf-2* longevity. It indicates that hypo-phosphorylated CDK-1 T179 may transmit a signal representing reduced DAF-2 pathway activity from the germline to the soma. Furthermore, results from our initial RNAi screen suggested dual roles of germline phosphoproteins in mediating the effects of IIS on reproduction and lifespan regulation. Notice that germline phosphoproteins are not necessarily germline-specific proteins. Techniques for tissue-specific mutagenesis of phosphosites are currently unavailable. Still, as a start, targeted protein degradation technologies, such as the AID method[22] used in the present study, were clearly helpful for testing hypotheses about tissue-specific signal transduction of IIS.

iFPS provides a systematic evaluation of functional phosphorylation in *C. elegans*

We thoroughly mapped and quantified the IIS-regulated phosphorylation changes in *C. elegans*. Our phosphoproteomic workflow yielded the largest-to-date phosphoproteome data set, almost doubling the size of previously available *C. elegans* database entries. To predict potentially functional phosphosites, we developed iFPS by integrating six types of sequence and structure features, including the number of predicted upstream kinase families (UKFs), the phosphorylation conservation (PhC), acetylation site co-occurrence (ASC) nearby the phosphosite, predicted relative surface accessibility (RSA), secondary structures (SSs) of the phosphosite, and the number of interacting domains and/or motifs (IDMs). Previous studies have correlated five of these features to functional phosphosites in yeast, mouse, rat, or human[23, 24, 25, 26]. Here, we evaluated and optimized the feature encoding for *C. elegans*.

Features such as the number of upstream individual kinases, absolute solvent accessibility (ASA), and co-occurrence of other PTM sites nearby phosphosites within ± 10 residues were incorporated in FunscoR, a recently released R package that functionally predicts human phosphosites[26]. The model performance of the FunscoR features trained on our benchmark data set were lower than those of the iFPS features (Fig. 2b and Supplementary Fig. 8d: number of upstream individual kinases AUC = 0.7465 versus UKF AUC = 0.7646; ASA AUC = 0.6518 versus RSA AUC = 0.6765; PTMs co-occurrence within ± 10 residues AUC = 0.5694 versus PTMs co-occurrence within ± 15 residues AUC = 0.5835). We measured PhC by the residue conservation score[27] and introduced a new feature IDM in our study. The iFPS model integrating six features is clearly superior to models built upon individual feature (Fig. 2b). We challenged the model training process on ten different sets of training data. The standard derivation of the AUC values were 0.0087, supporting the robustness of our method (Supplementary Fig. 8e).

Functional phosphosites positively correlated with scores of UKF, PhC and IDM (Supplementary Fig. 3a-c), while negatively correlated with scores of ASC and RSA (Supplementary Fig. 3d-e). These

were inherited in the final iFPS model, as reflected by the weight values of individual feature (1.7060 for UKF, 0.3302 for PhC, 0.0371 for IDM, -0.8108 for ASC, -5.1648 for RSA).

The negative correlation between functional phosphorylation and ASC or RSA seems counterintuitive. Phosphorylation might be induced by acetylation of the adjacent residue, as a study reported that acetylation of yeast histone H3 at K9 by GCN5 facilitates its phosphorylation at S10[28]. On the other hand, acetyl group is hydrophobic and might interfere with phosphorylation of nearby residues. For example, acetylation of human Tau at K259, K321 and K353 markedly inhibits its phosphorylation at S262, S324 and S356, respectively[29]. The negative weight value of ASC in our iFPS model indicated that functional phosphosites in *C. elegans* did not prefer to co-occur with nearby acetylation sites. As for RSA, it was proposed previously that easily accessible residues were more permissive substrates which could be non-specifically targeted by kinases and result in non-functional phosphosites[30]. Supporting this hypothesis, the negative weight value of RSA suggested that functional phosphosites in *C. elegans* tend to have lower accessibility.

Interestingly, functional phosphosites generally have lower scores of α-Helix (Supplementary Fig. 3f) and higher scores of β-strand and Coil (Supplementary Fig. 3g-h). However, the weight values of individual SS feature in the final iFPS model were 0.0578 for α-Helix, -1.6941 for β-strand, and 0.1959 for Coil, which suggested that the predicted functional phosphosites were not preferentially located at β-strands. The inconsistency may result from integration of multiple features and imply a context-specific property of the SS feature.

iFPS together with quantitative phosphoproteomics as a general approach to study functional phosphorylation events *in vivo*

iFPS scored 15,266 phosphosites in total. 50% of the true positive phosphosites were successfully recalled from the top 5% iFPS ranking, which is a ten-fold increase the propensity to hit a functional phosphosites from the overall background. Therefore, we considered a top 5% ranking as an initial cutoff for prioritizing the phosphosites regulated by *daf-2*. This prioritization strategy guided our follow-up experiments, which successfully demonstrated multiple mechanisms of lifespan

regulation by IIS at the level of phosphorylation. Notably, we also tested two phosphosites — EIF-5 pT376 and pS380 — that were among top 10-13% (Supplementary Fig. 6g-i). Our findings offer another form of validation for the utility of iFPS-based prioritization. Conventionally, EIF-5 pT376 and pS380 would almost certainly have been selected as candidates for functional studies. First, EIF-5 pS380 is regulated by *daf-2* (Supplementary Data 3). Second, EIF-5 pT376 is relatively conserved with a functional phosphosite S389 on human EIF5 (data from UniPort). Third, validating the EIF-5 phosphorylation may, like EIF-2α pS49, expand the knowledge on translational repression and lifespan regulation. However, mutational analysis suggested no significant effects for EIF-5 pT376 and pS380 on worm lifespan (Supplementary Fig. 6h-i). It was thus clear that our resource could help overcome the frequently encountered struggle with misleading "negative results" in attempts to validate findings from phosphoproteomic analyses.

iFPS scores, together with the *in vivo* phosphosites identified here, constitute a major resource for studies of phosphorylation and insulin signal transduction. *daf-2* and *daf-16* interacts with hundreds of genes that regulate lifespan. Our phosphoproteomic study uncovered 640 phosphorylation sites on proteins encoded by these IIS-related longevity genes (Supplementary Data 5). 95 of them ranked among top 5% of iFPS scoring, indicating potential functionality of the phosphorylation. Phosphosites on HSF-1 are among the top-ranking list, the phosphorylation levels of which were not yet quantified (Supplementary Data 1). *hsf-1* is required for the lifespan extension upon IIS reduction[31]. *daf-2* knockdown seems to induce unknown phosphorylation on HSF-1[32]. The reported phosphosites on human HSF1 are either constitutive or inducible and they either promote or repress HSF1 activity[33]. Targeted quantification of the iFPS ranked phosphosites will help characterize the functional phosphorylation on HSF-1 in future work.

a Overview of LC-MS/MS samples

Samples	Biological replicates	Technical replicates	Total # fractions (11-13 fractions /sample)
WT	3	2	78
daf-2	4	2	104
daf-16	4	2	88
daf-16; daf-2	4	2	92

b Overview of analysis results

	Identified (^{14}N-labeled)	Quantified
Phosphopeptides	22,536	
Phosphoisoforms	15,723	10,705
Phosphosites	15,443	10,343
Phosphoproteins	4,418	3,233

c Example showing the merge of phosphopeptides into phosphoisoforms

#	Phosphopeptide	Phosphoisoforms
1	TQSNLpSIPGSSSR	DAF-16 pS^{348}
2	PRTQSNLpSIPGSSSR	(merge 1 and 2)
3	TQpSNLpSIPGSSSR	DAF-16 pS^{345} pS^{348}
4	TQpSNLSIPGSSSR	DAF-16 pS^{345}

d

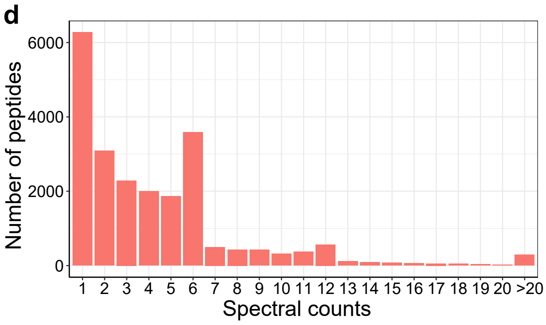

e

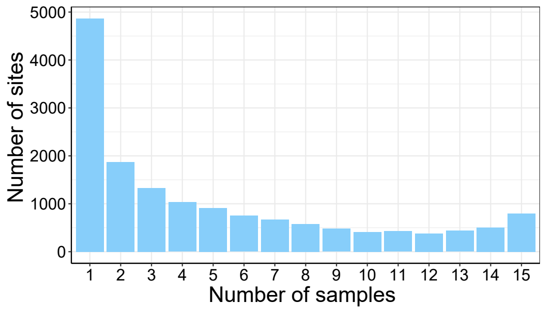

f

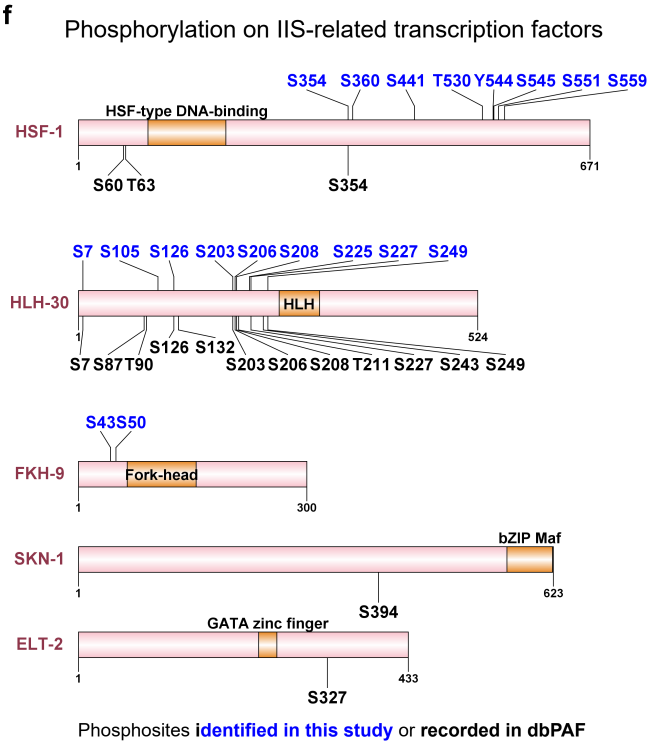

Supplementary Figure 1. Overview of this phosphoproteomic study, related to Fig. 1 and Supplementary Data 1.

a Numbers of MS samples surveyed. **b** Numbers of phosphorylation events identified and quantified. **c** Definition of phosphoisoforms. **d** Distribution of raw MS/MS spectral counts of the identified ^{14}N-labeled phosphopeptides. The mean spectral counts per phosphopeptide was 5.01. **e** Distribution of the identified phosphosites in 15 samples. The average number of samples per phosphosite was 4.72. **f** Phosphorylation on IIS-related transcription factors. Royal blue colors the phosphosites identified in this study, black colors the phosphosites recorded in dbPAF.

a

Examples for quantification of phosphopeptides in WT and the *daf-2* samples

Protein	Phosphopeptide$^{charge\ state}$	Quantitation value ($^{15}N/^{14}N$, ^{15}N-labeled peptides are internal reference across all samples)						
		WT_bio1	WT_bio2	WT_bio3	*daf-2*_bio1	*daf-2*_bio2	*daf-2*_bio3	*daf-2*_bio4
EIF-2α	EGMILLSELpS49R^{2+}	1.030303	–	–	0.439024	–	–	–
	LSEYNDKEGMILLSELpS49R^{2+}	–	–	–	–	–	0.80198	–
	LSEYNDKEGMILLSELpS49R^{3+}	–	–	–	0.402439	–	0.950495	–
	EGMILLSELpS49RR^{2+}	–	1.260417	0.513661	0.414634	0.625	0.792079	–
	LSEYNDKEGMILLSELpS49RR^{3+}	1.606061	1.520833	0.644809	–	–	–	–

Examples for quantification of phosphoisoforms in WT and the *daf-2* samples

Protein	Phosphoisoform	Quantitation value (median of $^{15}N/^{14}N$ ratios of phoshopeptide)						
		WT_bio1	WT_bio2	WT_bio3	*daf-2*_bio1	*daf-2*_bio2	*daf-2*_bio3	*daf-2*_bio4
EIF-2α	pS49	1.318182	1.390625	0.579235	0.414634	0.625	0.80198	–

Fold change of phosphorylation

Protein	Phosphoisoform	$\log_2(daf\text{-}2/WT)$
EIF-2α	pS49	1.07

$$\log_2(daf-2/WT) = \log_2\left[\frac{median(^{15}N/^{14}N)_{WT}}{median(^{15}N/^{14}N)_{daf-2}}\right]$$

^{15}N: internal reference
^{14}N: target

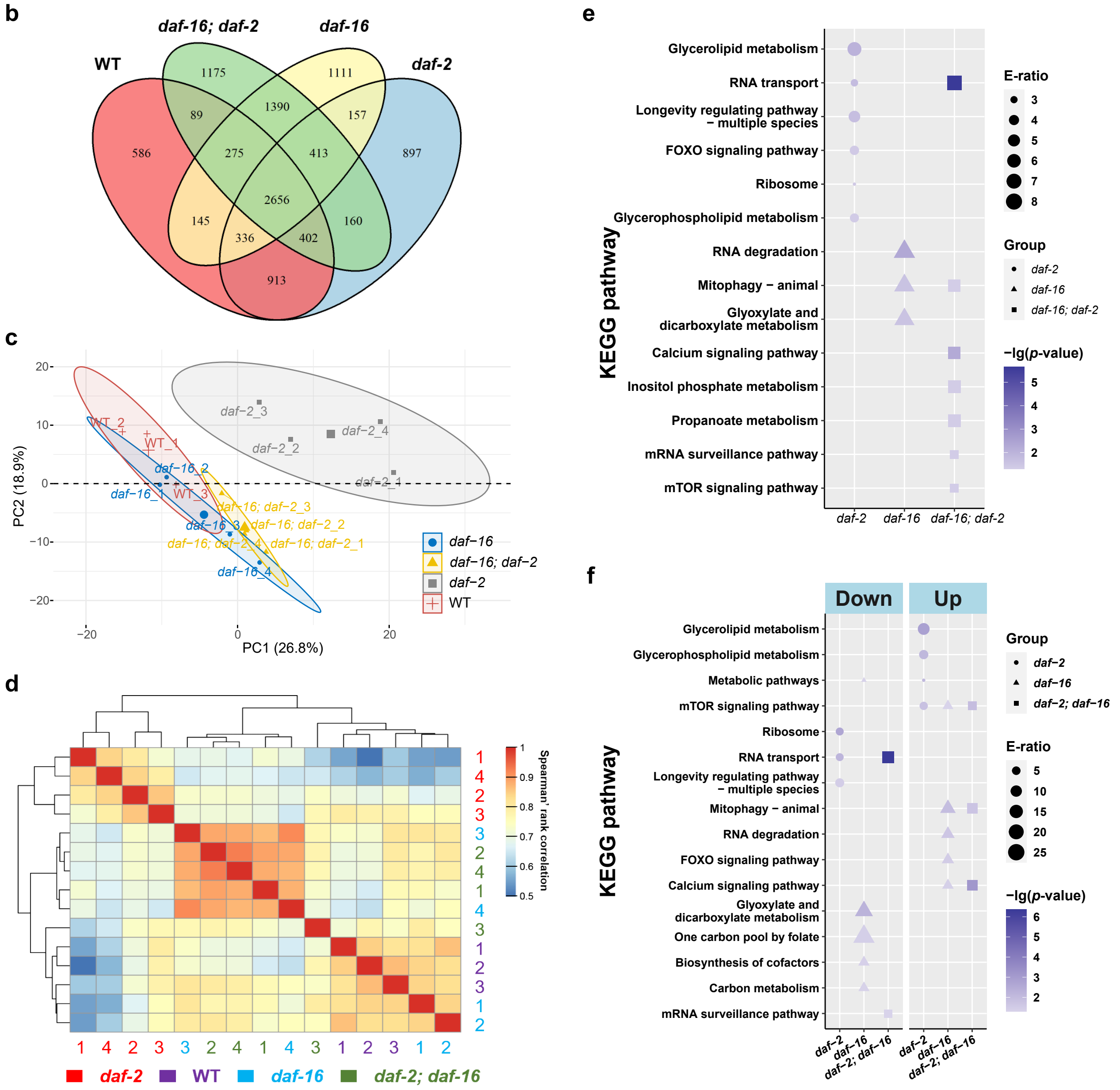

Supplementary Figure 2. Overview of the quantitation data, related to Fig. 1, Supplementary Fig. 1 and Supplementary Data 1.

a The quantitation data of EIF-2α phosphorylation as examples to illustrate how phosphorylation changes were calculated. **b** The Venn diagram shows the overlaps of the quantified phosphoisoforms among the four genotypes surveyed in this study. **c** Principal component analysis, which was based on 400 phosphoisoforms quantified in all 15 samples, suggested that the *daf-2* samples were different from others. **d** Heatmap shows the Spearman correlation coefficients pairwise calculated from thousands of phosphoisoforms quantified in each compared sample. The serial number of biological replicates of corresponding genotypes are presented as rows and columns. Unsupervised hierarchical clustering analysis demonstrated that the four samples of the long-lived *daf-2* mutant were clustered together, and separated from the WT, *daf-16*, and *daf-16; daf-2* samples. **e** The KEGG-based enrichment analysis of proteins differentially phosphorylated in the *daf*-2, *daf-16* or *daf-16; daf*-2 mutant. Phosphoisoforms quantified at least twice (meaning the number of $^{15}N/^{14}N$ ratios ≥ 2) in each genotype were subjected to comparison. Phosphoisoforms were determined as differentially phosphorylated if their fold change values increase or decrease above 1.5-factor when comparing each of the IIS mutants to WT. The same data set was used in Supplementary Fig. 2f. **f** The KEGG-based enrichment analysis of proteins with down-regulated or up-regulated phosphorylation in the *daf*-2, *daf-16* or *daf-16; daf-2* mutant.

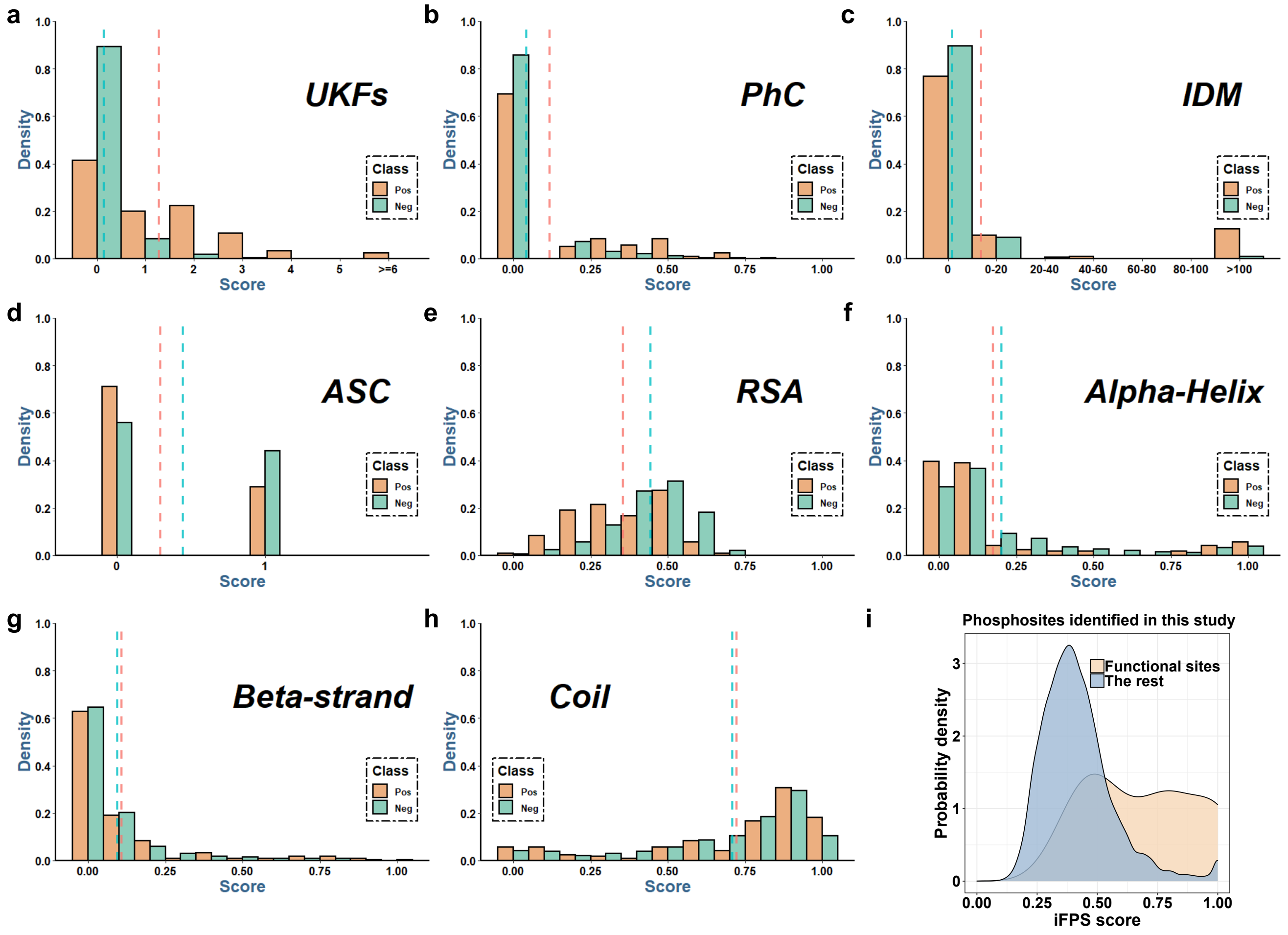

Supplementary Figure 3. Phosphorylation related features distinguished functional phosphosites in *C. elegans*, related to Fig. 2 and Supplementary Data 2.

a-h Distribution of scores evaluating phosphorylation related features for phosphosites in the positive data set (orange) and the negative data set (green). Features include upstream kinase families (UKFs) (**a**), phosphorylation conservation (PhC) (**b**), interacting domains and/or motifs (IDM) (**c**), acetylation site co-occurrence (ASC) (**d**), relative surface accessibility (RSA) (**e**), and secondary structures (SS) (**f-h**). **i** Distribution of the iFPS scores of phosphosites identified in this study. Yellow represents the known functional phosphosites among all identified phosphosites (*n* = 31), blue represents the rest of the scored phosphosites (*n* = 15,235).

MS-based targeted quantification of the endogenous peptide against the heavy labeled reference peptide

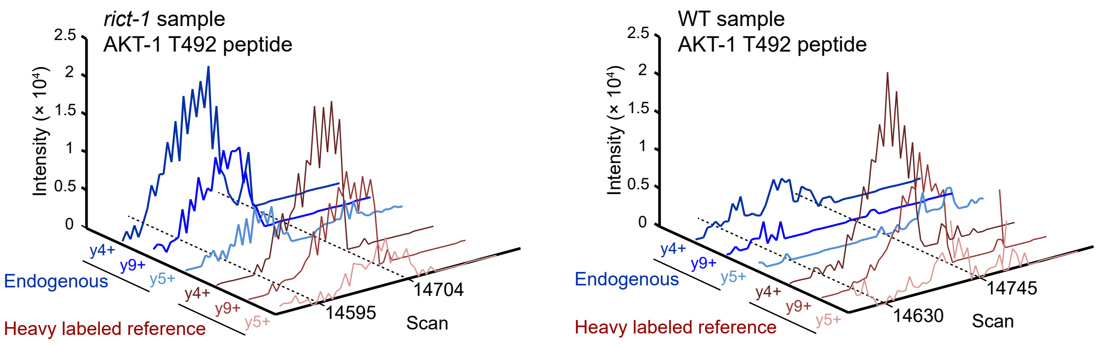

Endogenous peptide (EIEPPYKPNVQSETDTSYFDNEFTSQPVQLT[492]PPSR)
Heavy labeled reference peptide (EIEPPYKPNVQSETDTSYFDNEFTSQPVQLT[492]PPSR)

Supplementary Figure 4. AKT-1 T492 phosphorylation requires CeTORC2, related to Fig. 3 and Supplementary Data 6.

MS-based targeted quantification of the unphosphorylated AKT-1 T492 peptide from whole worm lysates. The extracted ion chromatograms of the $y4^{+}$, $y5^{+}$, and $y6^{+}$ fragment ions of the co-eluted endogenous or heavy labeled AKT-1 T492 peptide are shown. The peak intensities of the heavy isotope labeled reference peptide (red series) were steady in *rict-1(ft7)* and WT samples. The peaks from the endogenous peptide in *rict-1* sample (blue series) were as high as those from the reference peptide, whereas fragment ions from the endogenous peptide in WT sample were not detected. Scans between the dotted lines specify the co-elution time windows. Heavy labeled amino acid is colored in dark red with underline.

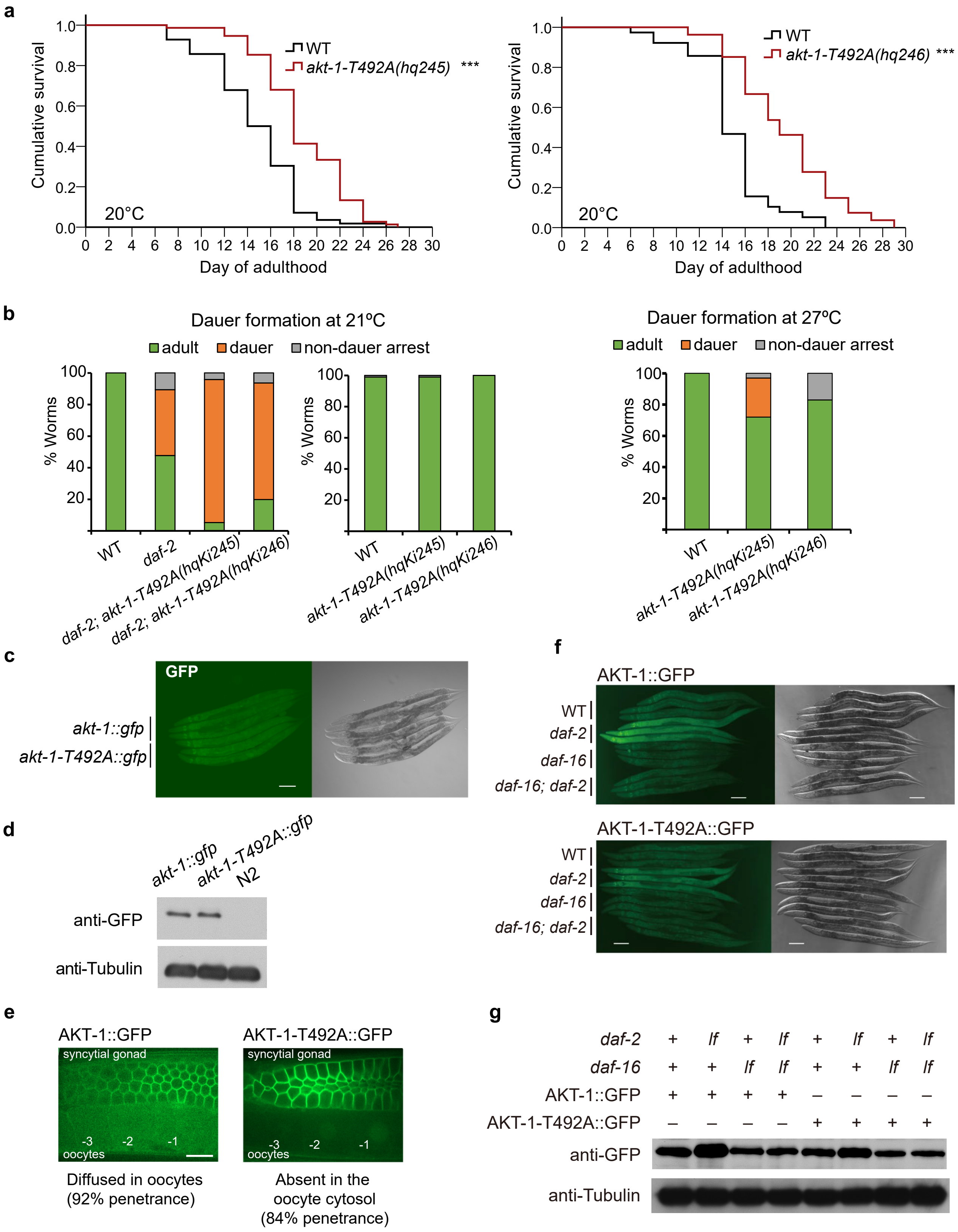
a
Cumulative survival
Day of adulthood
20°C
WT
akt-1-T492A(hq245) ***
akt-1-T492A(hq246) ***
b
Dauer formation at 21°C
Dauer formation at 27°C
adult
dauer
non-dauer arrest
% Worms
WT
daf-2
daf-2; akt-1-T492A(hqKi245)
daf-2; akt-1-T492A(hqKi246)
akt-1-T492A(hqKi245)
akt-1-T492A(hqKi246)
c
GFP
akt-1::gfp
akt-1-T492A::gfp
d
akt-1::gfp
akt-1-T492A::gfp
N2
anti-GFP
anti-Tubulin
e
AKT-1::GFP
AKT-1-T492A::GFP
syncytial gonad
-3
-2
-1
oocytes
Diffused in oocytes
(92% penetrance)
Absent in the
oocyte cytosol
(84% penetrance)
f
AKT-1::GFP
AKT-1-T492A::GFP
WT
daf-2
daf-16
daf-16; daf-2
g
daf-2
daf-16
AKT-1::GFP
AKT-1-T492A::GFP
anti-GFP
anti-Tubulin

Supplementary Figure 5. AKT-1 T492 phosphorylation regulates the subcellular localization of AKT-1 and compensates *daf-2* function in dauer formation, related to Fig. 3 and Supplementary Data 4.

a The *akt-1-T492A* mutation extended the worm lifespan at 20°C in FUdR-free conditions. ***$p < 0.001$, two-sided log-rank test, $n > 50$ per strain. See survival statistics in Supplementary Dataset 4. **b** The *akt-1-T492A* mutation enhanced the dauer formation phenotype in the *daf-2(e1370)* animals at 21°C. $n \geq 140$ animals per strain. More than 70% of the *akt-1-T492A* worms grew into adults at 27°C. $n > 120$ animals per strain. **c-d** AKT-1 T492A did not affect AKT-1 expression in WT animals. A GFP reporter was knocked in at the C-terminus of AKT-1 or AKT-1 T492A by CRISPR/Cas9. GFP fluorescence (**b**) and western blotting (**c**) showing no abundance difference between AKT-1 and AKT-1 T492A. Worms were sampled at adult day one. Scale bar: 100 μm. See biologically independent results ($n = 2$) in Source Data. **e** T492A mutation induced the nuclear localization of AKT-1 in proximal gonad. The gonad pictures of adult day one worms were taken under the same exposure condition by Spinning Disk Microscopy. Scale bar: 15 μm. Penetrance was calculated by scoring number of worms having the corresponding phenotypes under the Zeiss Axio Imager M1 microscope at 1000-fold magnification. $n = 35$ per strain. See Source Data for images acquired in ($n = 3$) independent experiments. **f-g** The *daf-2(e1370)* mutation induced the expression of AKT-1::GFP and AKT-1-T492A::GFP. The *daf-16(mu86)* mutation abolished such increase. Adult day one worms were sampled. Scale bar: 100 μm. See biologically independent results ($n = 2$) in Source Data.

a

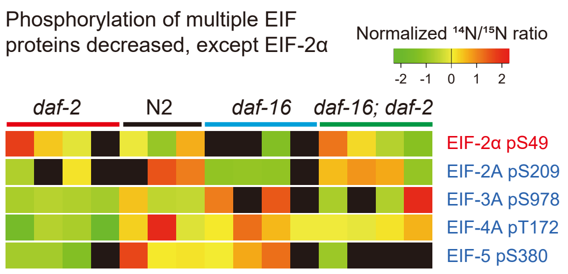

b

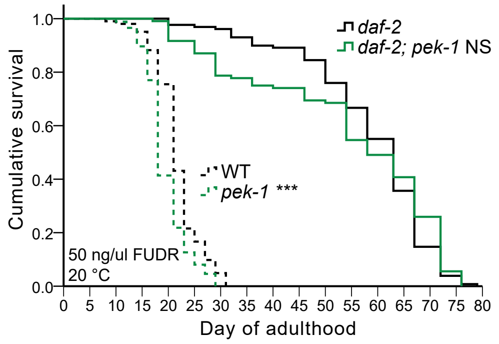

c

Peptides for targeted quantification analysis

EIF-2α pS49	LSEYNDKEGMILLSELpSRR
EIF-2α protein	LLEDIR

d

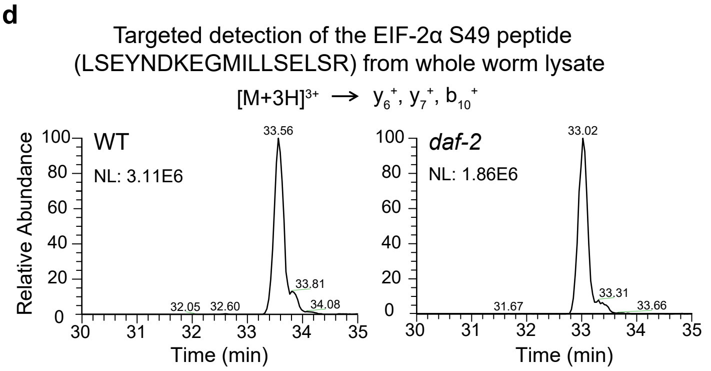

The EIF-2α pS49 peptide was undetectable in WT or *daf-2* lysate.

e

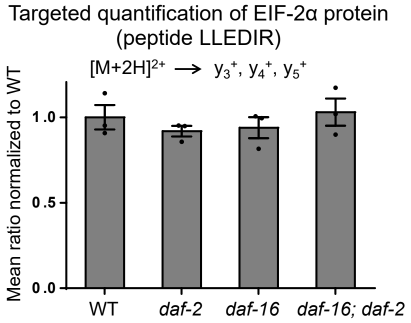

f

Increase of EIF-2α pS49 may be independent of *daf-16*

Quantification by omics	EIF-2α pS49
daf-2/WT	1.82
daf-2/*daf-16; daf-2*	0.95
daf-16; daf-2/WT	1.91

g

	Phosphosite	Ranking by iFPS score
EIF-5 (C37C3.2)	pT376	top 10.8%
	pS380	top 12.6%

	Phosphoisoform	*daf-2*/Ctrl
EIF-5 (C37C3.2)	pS380	0.73
	pT376; pS380	0.90

h

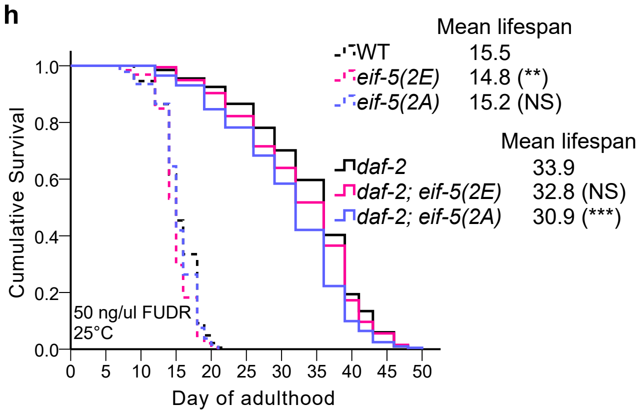

i

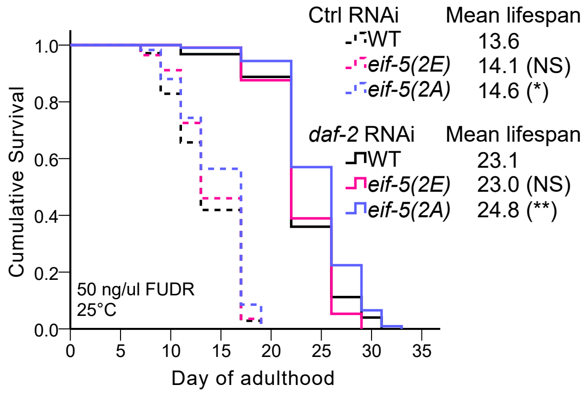

Supplementary Figure 6. Phosphorylation regulation of EIF-2α and other eukaryotic initiation factors in *C. elegans*, related to Fig. 4, Supplementary Data 3, 4, and 6.

a Phosphorylation of multiple EIF proteins decreased in the *daf-2* mutant (except for EIF-2α pS49). The *daf-2* regulated phosphoisoforms on EIF-2α (Y37E3.10), EIF-2A (E04D5.1), EIF-3A (EGL-45), EIF-4A (F53H1.1), and EIF-5 (C37C3.2) are shown. The heatmap presents the normalized $^{14}N/^{15}N$ ratios of each phosphoisoform, quantified across samples. **b** The *pek-1(ok275)* null mutants died significantly earlier than the WT worms, whereas no difference in lifespan was evident between the *daf-2(e1370)* and *daf-2(e1370); pek-1(ok275)* double mutants. **c** Synthesized peptides used in targeted mass spectrometry analysis. Isotopically labeled amino acids are underlined. **d** MS identified the EIF-2α S49 peptide but did not detect the EIF-2α pS49 peptide from whole worm lysates. Extracted ion chromatograms show the precursor ion of the endogenous EIF-2α S49 peptide. **e** Levels of EIF-2α protein were not significantly affected in the IIS mutants. Target peptide was quantified by MS against the cognate isotopically labeled peptide. Mean ratios were normalized to that in WT and showed no significant difference among the WT and IIS mutant worms ($n = 3$, two-tailed Student's t test). Error bars denote the SEM. **f** Hyper-phosphorylation on EIF-2α S49 in *daf-2* worms is DAF-16-independent. **g** The iFPS ranking and phosphorylation levels of phosphosites on EIF-5. **h-i** The endogenous EIF-5 T376 and S380 were mutated to T376E S380E (2E) or T376A S380A (2A) by CRISPR/Cas9. The *eif-5(2E)* and *eif-5(2A)* mutants exhibited either no, or little and inconsistent, alteration of the lifespan of WT or *daf-2* (*e1370* or RNAi) worms.

$^{*}p < 0.05$, $^{**}p < 0.01$, $^{***}p < 0.001$, NS not significant, two-sided log-rank test. See survival statistics in Supplementary Dataset 4.

a

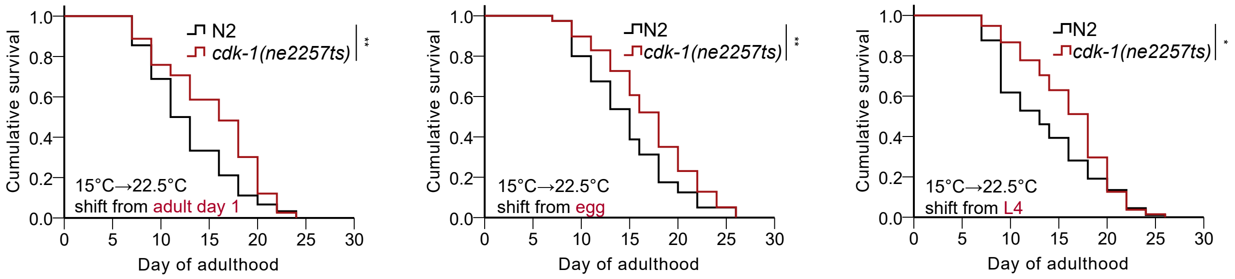

b

Protein	iFPS prioritized phosphosite	The *daf-2* regulated phosphoisoform	Phosphorylation *daf-2*/WT	Protein *daf-2*/WT#	Adult expression in germline or not?	Biological process
AAK-2	T597	pT597 pS601	1.47	2.17		
	S601	pT597 pS601	1.47			
	S570	pS570	1.44			
AKT-1	T492	pT492	2.21	2.44	√	
BYN-1	S46	pS46	1.37	0.86	√	Oogenesis
EIF-2α	S49	pS49	1.82	0.93	√	
MLT-3	S252	pS252	1.43	1.67	√	
AAK-2	S553	pS553	0.61	2.17		
CDK-1	T32	pT32 pY33	0.76	1.13	√	Cell cycle
	Y33	pT32 pY33; pY33	0.76; 0.51			
	T179	pT179	0.60			
CHK-2	S10	pS10 pS11	0.39	n.a.	√	Cell cycle
	S11	pS10 pS11	0.39			
DAF-16	S345	pS345 pS348	0.25	n.a.	√	
	S348	pS345 pS348	0.25			
EGL-45	S978	pS978	0.63	1.05	√	Oogenesis
IFA-1	S52	pS49 pS52	0.73	0.56		Cell cycle
LIG-1	S732	pS732 pS734	0.45	1.3	√	Cell cycle
NMY-2	S211	pS211	0.73	1.07	√	Oogenesis
PDHA-1	S287	pS287	0.76	0.97	√	Oogenesis
PPFR-1	S1137	pS1137	0.71	n.a.	√	Cell cycle
PQN-59	S255	pS253 pS255	0.68	0.74	√	
PUF-3	S56	pS56	0.23	n.a.	√	Cell cycle
T04A8.8	S245	pS245	0.67	0.77	√	
TOST-1	S94	pS94	0.42	0.93	√	Oogenesis

c

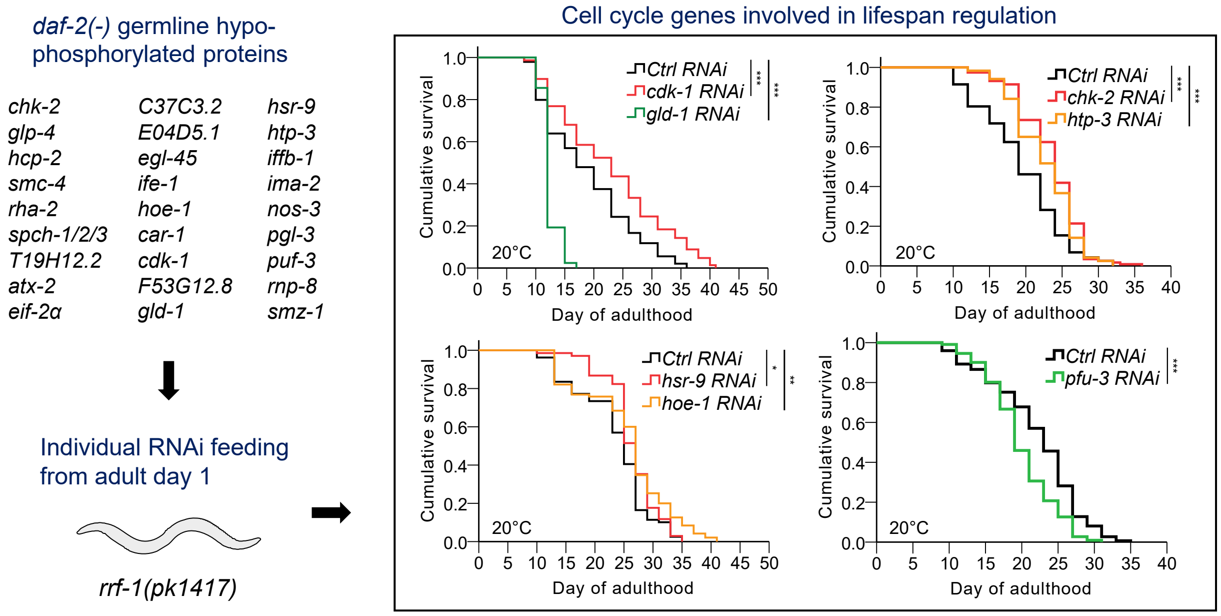

Supplementary Figure 7. Germline proteins which were hypo-phosphorylated in *daf-2* regulate lifespan in *C. elegans*, related to Fig. 5 and Supplementary Data 4.

a Inactivation of CDK-1 in early life or adult stage significantly extended lifespan. Worms were cultured at 15°C and shifted to 22.5°C from adult day one, freshly laid egg, or L4 stage. **b** The iFPS prioritized phosphosites shown in Fig. 2d reside on proteins commonly expressed in the germline. 11 proteins are known to function in cell cycle or oogenesis. Expression and phenotype data derived from WormBase. # proteomics data derived from *Walther et al., 2015*[1]. **c** Screening for lifespan regulators among the germline proteins that were hypo-phosphorylated in *daf-2* worms. An individual RNAi clone was fed to the *rrf-1(pk1417)* mutant from adult day one at 20°C. Lifespan results that showed significant changes upon target gene knockdown in the initial screens were confirmed in independent lifespan assays.

*$p < 0.05$, **$p < 0.01$, ***$p < 0.001$, two-sided log-rank test. See survival statistics in Supplementary Dataset 4.

a

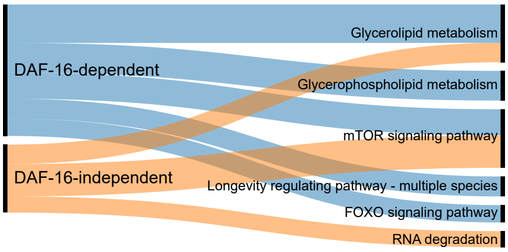

c

Enrichment method (Cutoff)	Hypergeometric test ($p < 0.05$)	KSEA (top 27)	KSEA ($p < 0.05$)
Lifespan-related kinases	10	4	9
Total enriched kinases	27	27	50

b

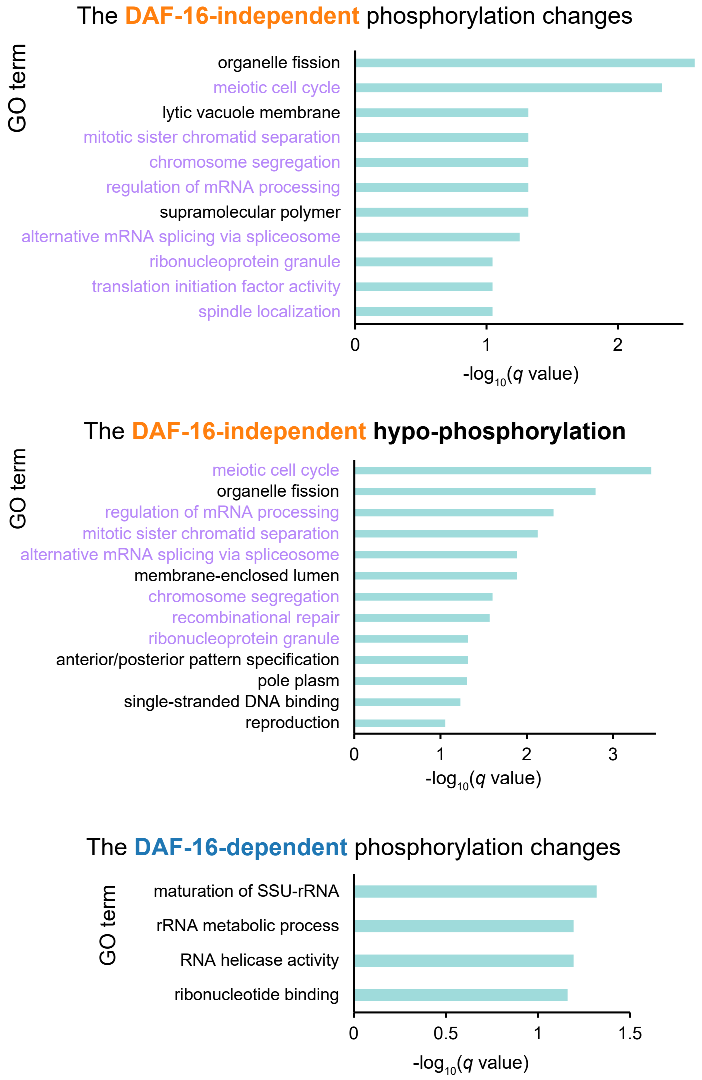

d

e

f

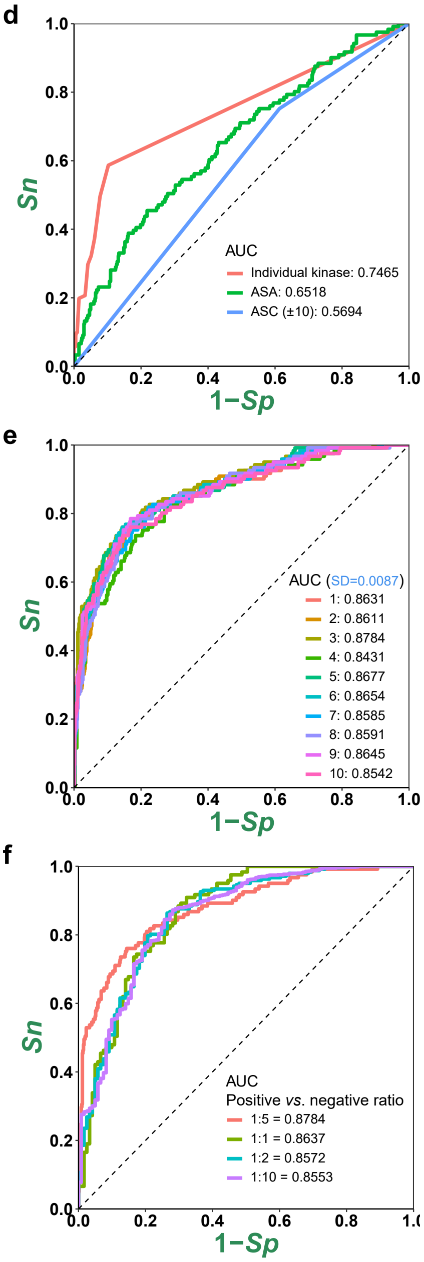

Supplementary Figure 8. related to Discussion, Methods and Supplementary Data 2-3.
a The KEGG-based enrichment results of the DAF-16-dependent or independent phosphorylation changes in the *daf-2* mutant. The significantly enriched pathways (E-ratio >1, $p < 0.05$) are shown. **b** The GO enrichment results (q value < 0.1) of the DAF-16-dependent and DAF-16-independent phosphorylation changes (the entire group or the hypo-phosphorylated group) in the *daf-2* mutant. GO terms that may link to cell cycle or mRNA translation are highlighted in purple. Enrichment analysis were conducted via online tools in WormBase[2]. **c** Kinase substrate enrichment analysis against the phosphorylation changes in the *daf-2* mutant. 10 of the 27 enriched kinases (hypergeometric test, $p < 0.05$) were reported to regulate lifespan. 9 of the 50 KSEA-enriched kinases (kinase-substrate relation number m ≥ 5, $p < 0.05$) were known lifespan regulators, but only 4 of them ranked among top 27. The hypo-phosphorylated substrates in the *daf-2* mutant were analyzed by the "hypergeometric test"-based enrichment. Phosphorylation fold changes (*daf-2/Ctrl)* were used to calculate the KSEA scores. See the Source data file. **d** The ROC curves of models with single feature including individual kinase, ASA and ASC (±10). **e** The ROC curves of ten times training of iFPS. **f** Prediction performance comparison between models constructed with different ratios of positive and negative data. Sn sensitivity, Sp specificity.

Supplementary Table 1. Abbreviations used in this article

Abbreviation	Definition
AID	auxin-induced protein degradation
ASC	acetylation site co-occurrence
AUC	area under the curve
CI	confidence interval
CK2	casein kinase 2
dbPAF	database of phosphosites in human, animals and fungi
EIF	eukaryotic initiation factor
IDM	interacting domains and/or motif
iFPS	inference of functional phosphosites
IGF-1	Insulin-like growth factor 1
iGPS	*in vivo* Group-based Prediction System
IIS	Insulin/IGF-1 signaling
LC-MS/MS	liquid chromatography-tandem mass spectrometry
lf	loss-of-function
LiRP	lifespan-related phosphosite
PhC	phosphorylation conservation
PI3K	phosphatidylinositol-3-OH kinase
PPI	protein-protein interaction
PROTAC	proteolysis-targeting chimera
PTM	post-translational modification
RCS	residue conservation score
RNAi	RNA interference
RSC	relative surface accessibility
S/T	serine/threonine
SS	secondary structure
ssKSR	site-specific kinase-substrate relation
TBB	4,5,6,7-tetrabromo-1H-benzotriazole
TF	transcription factor
UKF	upstream kinase family
WT	wild-type

Supplementary Table 2. *C. elegans* strains used in this study. All worms assayed were hermaphrodites. The usage of larvae or adult worms were specified in experiments.

C. elegans Strain	Source	Method
MQD854: N2 wild-type	This paper	outcross x5
MQD855: *daf-2(e1370ts) III*	This paper	outcross x5
MQD856: *daf-16(mu86) I*	This paper	outcross x5
MQD857: *daf-16(mu86) I; daf-2(e1370ts) III*	This paper	outcross x5
MQD1543: *daf-16(hqKi23[daf-16::gfp::6his]) I*	This paper	CRISPR/Cas9
MQD2333: *daf-16(hqKi23) I; akt-1(hqKi245) V*	This paper	cross
MQD2334: *daf-16(hqKi23) I; akt-1(hqKi246) V*	This paper	cross
MQD2114: *akt-1(hqKi245[akt-1a-T492A]) V*	This paper	CRISPR/Cas9
MQD2115: *akt-1(hqKi246[akt-1a-T492A]) V*	This paper	CRISPR/Cas9
MQD2681: *akt-1(hqKi475[akt-1::GFP]) V*	This paper	CRISPR/Cas9
MQD2919: *daf-2(e1370ts) III; akt-1(hqKi475[akt-1::GFP]) V*	This paper	cross
MQD2743: *daf-16(mu86) I; akt-1(hqKi475[akt-1::GFP]) V*	This paper	cross
MQD2744: *daf-16(mu86) I; daf-2(e1370ts) III; akt-1(hqKi475[akt-1::GFP]) V*	This paper	cross
MQD2688: *akt-1(hqKi246hqKi476[akt-1-T492A::gfp]) V*	This paper	CRISPR/Cas9
MQD2921: *daf-2(e1370ts) III; akt-1(hqKi246hqKi476[akt-1-T492A::gfp]) V*	This paper	cross
MQD2745: *daf-16(mu86) I; akt-1(hqKi246hqKi476[akt-1-T492A::gfp]) V*	This paper	cross
MQD2746: *daf-16(mu86) I; daf-2(e1370ts) III; akt-1(hqKi246hqKi476[akt-1-T492A::gfp]) V*	This paper	cross
KQ1366: *rict-1(ft7) II*	A gift from Dr. Hong Zhang	N/A
MQD1516: *gcn-2(ok871) II*	This paper	outcross x4
MQD1517: *gcn-2(ok886) II*	This paper	outcross x4
MQD1554: *gcn-2(ok871) II; daf-2(e1370ts) III*	This paper	cross
MQD1555: *gcn-2(ok886) II; daf-2(e1370ts) III*	This paper	cross
MQD1600: *pek-1(ok275) X*	This paper	outcross x4
MQD1603: *eif-2α(hqKi188[eif-2α-S49A]) I*	This paper	CRISPR/Cas9
MQD1605: *eif-2α(hqKi190[eif-2α-S49A]) I*	This paper	CRISPR/Cas9

MQD1606: *daf-2(e1370ts) III; pek-1(ok275) X*	This paper	cross
MQD1616: *eif-2α(hqKi188[eif-2α-S49A]) I; daf-2(e1370ts) III*	This paper	cross
MQD1617: *eif-2α(hqKi190[eif-2α-S49A]) I; daf-2(e1370ts) III*	This paper	cross
MQD1355: *hqIs404[peif-2α::eif-2α-S49A::gfp, pRF4]*	This paper	outcross x4
MQD1359: *hqIs406[peif-2α::eif-2α-S49A::gfp, pRF4]*	This paper	outcross x4
MQD1618: *atf-5(hq35) X*	This paper	CRISPR/Cas9
MQD1619: *atf-5(hq36) X*	This paper	CRISPR/Cas9
MQD1644: *daf-2(e1370ts) III; atf-5(hq35) X*	This paper	cross
MQD1645: *daf-2(e1370ts) III; atf-5(hq36) X*	This paper	cross
MQD2007: *eif-5(hqKi162[C37C3.2-T376E-S380E]) V*	This paper	CRISPR/Cas9
MQD2008: *eif-5(hqKi427[C37C3.2-T376E-S380E]) V*	This paper	CRISPR/Cas9
MQD2020: *eif-5(hqKi168[C37C3.2-T376A-S380A]) V*	This paper	CRISPR/Cas9
MQD2021: *eif-5(hqKi169[C37C3.2-T376A-S380A]) V*	This paper	CRISPR/Cas9
MQD2041: *daf-2(e1370ts) III; eif-5(hqKi162[C37C3.2-T376E-S380E]) V*	This paper	cross
MQD2042: *daf-2(e1370ts) III; eif-5(hqKi168[C37C3.2-T376A-S380A]) V*	This paper	cross
MAH23: *rrf-1(pk1417) I*	A gift from Dr. Di Chen	N/A
WM99: *cdk-1(ne2257ts) III*	Caenorhabditis Genetics Center	N/A
MQD2324: *rrf-1(pk1417) daf-16(mu86) I*	This paper	cross
MQD2330: *daf-16(mu86) I; cdk-1(ne2257ts) III*	This paper	cross
NR222: *rde-1(ne219)V; kzIs9*	Caenorhabditis Genetics Center	N/A
VP303: *rde-1(ne219)V; kzIs7*	Caenorhabditis Genetics Center	N/A
CA1199: *unc-119(ed3) III; ieSi38[sun-1p::TIR1::mRuby::sun-1 3'UTR+ Cbr-unc-119(+)] IV*	Caenorhabditis Genetics Center	N/A
MQD2428: *daf-2(hqKi363[Pdaf-2::daf-2::degron::mNeonGreen]) III*	This paper	CRISPR/Cas9
MQD2375: *daf-2(hqKi363) III; ieSi38 IV*	This paper	CRISPR/Cas9

MQD2483: *wee-1.3(hqKi412[mNeonGreen::degron::wee-1.3]) II; unc-119(ed3) III; ieSi38 IV*	This paper	CRISPR/Cas9
MQD2484: *wee-1.3(hqKi413[mNeonGreen::degron::wee-1.3]) II; unc-119(ed3) III; ieSi38 IV*	This paper	CRISPR/Cas9
MQD2535: *wee-1.3(hqKi412) II; daf-2(hqKi363) III; ieSi38 IV*	This paper	cross
MQD2536: *wee-1.3(hqKi413) II; daf-2(hqKi363) III; ieSi38 IV*	This paper	cross

References

1. Son HG, Seo M, Ham S, Hwang W, Lee D, An SW*, et al.* RNA surveillance via nonsense-mediated mRNA decay is crucial for longevity in daf-2/insulin/IGF-1 mutant C. elegans. *Nature communications* 2017, **8:** 14749.

2. Walther DM, Kasturi P, Zheng M, Pinkert S, Vecchi G, Ciryam P*, et al.* Widespread Proteome Remodeling and Aggregation in Aging C. elegans. *Cell* 2015, **161**(4)**:** 919-932.

3. Williams GC. Pleiotropy, natural selection, and the evolution of senescence. *Evolution* 1957, **11:** 398-411.

4. Wiredja DD, Koyuturk M, Chance MR. The KSEA App: a web-based tool for kinase activity inference from quantitative phosphoproteomics. *Bioinformatics* 2017, **33**(21)**:** 3489-3491.

5. Essers PB, Nonnekens J, Goos YJ, Betist MC, Viester MD, Mossink B*, et al.* A Long Noncoding RNA on the Ribosome Is Required for Lifespan Extension. *Cell reports* 2015, **10**(3)**:** 339-345.

6. Sonenberg N, Hinnebusch AG. Regulation of translation initiation in eukaryotes: mechanisms and biological targets. *Cell* 2009, **136**(4)**:** 731-745.

7. Rousakis A, Vlassis A, Vlanti A, Patera S, Thireos G, Syntichaki P. The general control nonderepressible-2 kinase mediates stress response and longevity induced by target of rapamycin inactivation in Caenorhabditis elegans. *Aging cell* 2013, **12**(5)**:** 742-751.

8. D'Aniello C, Fico A, Casalino L, Guardiola O, Di Napoli G, Cermola F*, et al.* A novel autoregulatory loop between the Gcn2-Atf4 pathway and l-Proline metabolism controls stem cell identity. *Cell death and differentiation* 2015, **22**(7)**:** 1234.

9. Ye J, Kumanova M, Hart LS, Sloane K, Zhang H, De Panis DN*, et al.* The GCN2-ATF4 pathway is critical for tumour cell survival and proliferation in response to nutrient deprivation. *The EMBO journal* 2010, **29**(12)**:** 2082-2096.

10. Henis-Korenblit S, Zhang P, Hansen M, McCormick M, Lee SJ, Cary M*, et al.* Insulin/IGF-1 signaling mutants reprogram ER stress response regulators to promote longevity. *Proceedings of the National Academy of Sciences of the United States of America* 2010, **107**(21)**:** 9730-9735.

11. Darnell AM, Subramaniam AR, O'Shea EK. Translational Control through Differential Ribosome Pausing during Amino Acid Limitation in Mammalian Cells. *Molecular cell* 2018, **71**(2)**:** 229-243 e211.

12. Dong J, Qiu H, Garcia-Barrio M, Anderson J, Hinnebusch AG. Uncharged tRNA activates GCN2 by displacing the protein kinase moiety from a bipartite tRNA-binding domain. *Molecular cell* 2000, **6**(2)**:** 269-279.

13. Depuydt G, Xie F, Petyuk VA, Shanmugam N, Smolders A, Dhondt I*, et al.* Reduced insulin/insulin-like growth factor-1 signaling and dietary restriction inhibit translation but preserve muscle mass in Caenorhabditis elegans. *Molecular & cellular proteomics : MCP* 2013, **12**(12)**:** 3624-3639.

14. Gao AW, Smith RL, van Weeghel M, Kamble R, Janssens GE, Houtkooper RH. Identification of key pathways and metabolic fingerprints of longevity in C. elegans. *Experimental gerontology* 2018, **113:** 128-140.

15. Stout GJ, Stigter EC, Essers PB, Mulder KW, Kolkman A, Snijders DS*, et al.* Insulin/IGF-1-mediated longevity is marked by reduced protein metabolism. *Molecular systems biology* 2013, **9:** 679.

16. Apfeld J, Kenyon C. Cell nonautonomy of C. elegans daf-2 function in the regulation of diapause and life span. *Cell* 1998, **95**(2)**:** 199-210.

17. Libina N, Berman JR, Kenyon C. Tissue-specific activities of C. elegans DAF-16 in the regulation of lifespan. *Cell* 2003, **115**(4)**:** 489-502.

18. Zhang P, Judy M, Lee SJ, Kenyon C. Direct and indirect gene regulation by a life-extending FOXO protein in C. elegans: roles for GATA factors and lipid gene regulators. *Cell metabolism* 2013, **17**(1)**:** 85-100.

19. Hsin H, Kenyon C. Signals from the reproductive system regulate the lifespan of C. elegans. *Nature* 1999, **399**(6734)**:** 362-366.

20. Michaelson D, Korta DZ, Capua Y, Hubbard EJ. Insulin signaling promotes germline proliferation in C. elegans. *Development* 2010, **137**(4)**:** 671-680.

21. Moll L, Roitenberg N, Bejerano-Sagie M, Boocholez H, Carvalhal Marques F, Volovik Y*, et al.* The insulin/IGF signaling cascade modulates SUMOylation to regulate aging and proteostasis in Caenorhabditis elegans. *eLife* 2018, **7**.

22. Zhang L, Ward JD, Cheng Z, Dernburg AF. The auxin-inducible degradation (AID) system enables versatile conditional protein depletion in C. elegans. *Development* 2015, **142**(24)**:** 4374-4384.

23. Niu S, Wang Z, Ge D, Zhang G, Li Y. Prediction of functional phosphorylation sites by incorporating evolutionary information. *Protein & cell* 2012, **3**(9)**:** 675-690.

24. Xiao Q, Miao B, Bi J, Wang Z, Li Y. Prioritizing functional phosphorylation sites based on multiple feature integration. *Scientific reports* 2016, **6:** 24735.

25. Beltrao P, Albanese V, Kenner LR, Swaney DL, Burlingame A, Villen J*, et al.* Systematic functional prioritization of protein posttranslational modifications. *Cell* 2012, **150**(2)**:** 413-425.

26. Ochoa D, Jarnuczak AF, Vieitez C, Gehre M, Soucheray M, Mateus A*, et al.* The functional landscape of the human phosphoproteome. *Nature biotechnology* 2020, **38**(3)**:** 365-373.

27. Minguez P, Parca L, Diella F, Mende DR, Kumar R, Helmer-Citterich M*, et al.* Deciphering a global network of functionally associated post-translational modifications. *Molecular systems biology* 2012, **8:** 599.

28. Nowak SJ, Corces VG. Phosphorylation of histone H3: a balancing act between chromosome condensation and transcriptional activation. *Trends in genetics : TIG* 2004, **20**(4)**:** 214-220.

29. Carlomagno Y, Chung DC, Yue M, Castanedes-Casey M, Madden BJ, Dunmore J*, et al.* An acetylation-phosphorylation switch that regulates tau aggregation propensity and function. *The Journal of biological chemistry* 2017, **292**(37)**:** 15277-15286.

30. Landry CR, Levy ED, Michnick SW. Weak functional constraints on phosphoproteomes. *Trends in genetics : TIG* 2009, **25**(5)**:** 193-197.

31. Hsu AL, Murphy CT, Kenyon C. Regulation of aging and age-related disease by DAF-16 and heat-shock factor. *Science* 2003, **300**(5622)**:** 1142-1145.

32. Chiang WC, Ching TT, Lee HC, Mousigian C, Hsu AL. HSF-1 regulators DDL-1/2 link insulin-like signaling to heat-shock responses and modulation of longevity. *Cell* 2012, **148**(1-2)**:** 322-334.

33. Xu YM, Huang DY, Chiu JF, Lau AT. Post-translational modification of human heat shock factors and their functions: a recent update by proteomic approach. *Journal of proteome research* 2012, **11**(5)**:** 2625-2634.

34. Angeles-Albores D, Lee R, Chan J, Sternberg P. Two new functions in the WormBase Enrichment Suite. *microPublication biology* 2018, **2018**.
