# Supplementary material for: Insulin signaling regulates longevity through protein phosphorylation in Caenorhabditis elegans
Source: Nat Commun. 2021 Jul 27;12:4568. doi: 10.1038/s41467-021-24816-z (PMC8316574; doi:10.1038/s41467-021-24816-z)
Supplement: Supplementary file 9 — Description of Additional Supplementary Files [file 41467_2021_24816_MOESM9_ESM.pdf]

Description of additional supplementary information

File: Supplementary Data 1.

Description: The quantified phosphoisoforms and identified phosphosites in this study.

File: Supplementary Data 2.

Description: Benchmark data sets for iFPS and the scores of the *C. elegans* phosphosites.

File: Supplementary Data 3.

Description: The *daf-2* regulated phosphoisoforms with the iFPS scoring and the DAF-16-dependent/independent subsets.

File: Supplementary Data 4.

Description: The *C. elegans* lifespan data.

File: Supplementary Data 5.

Description: Phosphosites on the IIS-related longevity proteins.

File: Supplementary Data 6.

Description: Ions measured by LC-MS/MS in the targeted quantification assays.
