# Supplementary material for: Insulin signaling regulates longevity through protein phosphorylation in Caenorhabditis elegans
Source: Nat Commun. 2021 Jul 27;12:4568. doi: 10.1038/s41467-021-24816-z (PMC8316574; doi:10.1038/s41467-021-24816-z)
Supplement: Supplementary file 10 — Reporting Summary [file 41467_2021_24816_MOESM10_ESM.pdf]

nature research

Corresponding author(s): Yu Xue and Meng-Qiu Dong

Last updated by author(s): Jun 10, 2021

Reporting Summary

Nature Research wishes to improve the reproducibility of the work that we publish. This form provides structure for consistency and transparency in reporting. For further information on Nature Research policies, see our Editorial Policies and the Editorial Policy Checklist.

Statistics

For all statistical analyses, confirm that the following items are present in the figure legend, table legend, main text, or Methods section.

n/a	Confirmed	
☐	☒	The exact sample size (*n*) for each experimental group/condition, given as a discrete number and unit of measurement
☐	☒	A statement on whether measurements were taken from distinct samples or whether the same sample was measured repeatedly
☐	☒	The statistical test(s) used AND whether they are one- or two-sided<br>*Only common tests should be described solely by name; describe more complex techniques in the Methods section.*
☒	☐	A description of all covariates tested
☒	☐	A description of any assumptions or corrections, such as tests of normality and adjustment for multiple comparisons
☐	☒	A full description of the statistical parameters including central tendency (e.g. means) or other basic estimates (e.g. regression coefficient) AND variation (e.g. standard deviation) or associated estimates of uncertainty (e.g. confidence intervals)
☐	☒	For null hypothesis testing, the test statistic (e.g. *F*, *t*, *r*) with confidence intervals, effect sizes, degrees of freedom and *P* value noted<br>*Give P values as exact values whenever suitable.*
☒	☐	For Bayesian analysis, information on the choice of priors and Markov chain Monte Carlo settings
☒	☐	For hierarchical and complex designs, identification of the appropriate level for tests and full reporting of outcomes
☒	☐	Estimates of effect sizes (e.g. Cohen's *d*, Pearson's *r*), indicating how they were calculated

Our web collection on statistics for biologists contains articles on many of the points above.

Software and code

Policy information about availability of computer code

Data collection

MS/MS data were acquired with Xcalibur 4.0 (Thermo Fisher Scientific). The C. elegans protein and phenotype databases were derived from WormBase release WS233 (ftp://ftp.wormbase.org/pub/wormbase/releases/WS233/), WormBase release WS275 (ftp://ftp.wormbase.org/pub/wormbase/releases/WS275/), or UniProt release 2015_01 (https://ftp.uniprot.org/pub/databases/uniprot/previous_releases/release-2015_01/). The benchmark data sets of iFPS were collected from PubMed and dbPAF (http://dbpaf.biocuckoo.org/). The pre-compiled domain-domain and domain-motif interactions were downloaded from the database of three-dimensional interacting domains (3did). Images of western blots were recorded by LI-COR Odyssey Infrared Imaging System. Images of worms were captured and processed by Axiovision Rel. 4.7 software (Carl Zeiss Ltd.) or Volocity Demo 6.3 (PerkinElmer).

Data analysis

1. Phosphoproteomics raw data were analyzed by RawXtract 1.9.9.2, ProLuCID, DTASelect2, PhosphoRS, and pQuant.
2. iGPS 1.0 (http://igps.biocuckoo.org/) was used to predict site-specific kinase-substrate relations (ssKSRs). BLAST (version 2.2.31) were used to detect orthologs of worm phosphoproteins in other eukaryotes. MUSCLE were adopted to multi-align protein sequence. GPS-PAIL 2.0 (http://pail.biocuckoo.org/) was used to predict the substrates of histone acetyltransferases. NetSurfP v1.1 (http://www.cbs.dtu.dk/services/NetSurfP/) was used for the prediction of relative surface accessibility (RSA) and second structure of phosphoproteins. The hmmsearch program in HMMER v3.1b2 was used to predict domains in phosphoproteins. Java package Weka 3.8 were used in developing iFPS.
3. MS based target quantification were performed using Xcalibur 2.2 SP1.48 and pLabel (version 2.4). Images were quantified with Image Studio Lite Ver 4.0 (LI-COR). Tissue expression prediction scores were calculated based on an interactive webserver (http://worm.princeton.edu). R 3.5.0, Excel 2013 and SPSS version 20 were used in statistical analysis.

For manuscripts utilizing custom algorithms or software that are central to the research but not yet described in published literature, software must be made available to editors and reviewers. We strongly encourage code deposition in a community repository (e.g. GitHub). See the Nature Research guidelines for submitting code & software for further information.

Data

Policy information about availability of data

All manuscripts must include a data availability statement. This statement should provide the following information, where applicable:

- Accession codes, unique identifiers, or web links for publicly available datasets
- A list of figures that have associated raw data
- A description of any restrictions on data availability

1. The MS/MS raw data sets for phosphoproteomics and target quantitation in this study have been deposited to the ProteomeXchange Consortium via the iProX partner repository with the dataset identifier PXD020440 (https://www.iprox.cn/page/project.html?id=IPX0002300000).
2. The C. elegans databases used in this study were WormBase release WS233 (ftp://ftp.wormbase.org/pub/wormbase/releases/WS233/), WormBase release WS275 (ftp://ftp.wormbase.org/pub/wormbase/releases/WS275/), and UniProt release 2015_01 (https://ftp.uniprot.org/pub/databases/uniprot/previous_releases/release-2015_01/).
3. Phosphoproteomics data were summarized in Supplementary Data 1 and 3. Target quantification data were shown in Supplementary Data 6. iFPS scoring data were presented in Supplementary Data 2 and 5. Lifespan data were collected in Supplementary Data 4.
4. All relevant data are available from the corresponding authors on reasonable request.
5. The source data of figures were provided in the "Source data.xlsx" file.

Field-specific reporting

Please select the one below that is the best fit for your research. If you are not sure, read the appropriate sections before making your selection.

☒ Life sciences ☐ Behavioural & social sciences ☐ Ecological, evolutionary & environmental sciences

For a reference copy of the document with all sections, see nature.com/documents/nr-reporting-summary-flat.pdf

Life sciences study design

All studies must disclose on these points even when the disclosure is negative.

Sample size	Sample size, biological replicates, and statistical methods were chosen based on previous studies reported in the literature.<br>For quantitative phosphoproteomics: PMID 23820781, 25373143, 26392051.<br>For target quantification by mass spec: PMID 26633379.<br>For immunoblotting analysis: PMID 18413715, 22719267, 23805378.<br>For lifespan analysis: PMID 23805378.<br>For dauer formation analysis: PMID 19249087.<br>For polyribosome profiling: PMID 23820781.<br>For miscroscopy: PMID 30773782, 26115433.
Data exclusions	In lifespan assays, worms that had internally hatched larvae (‘bagged’) or ruptured vulvae (‘exploded’) or crawled off the agar surface were censored.
Replication	Phosphoproteomics data of the wild-type and IIS mutant C. elegans were obtained with at least three biological replicates. MS based target quantification were assayed with at least two biological replicates. All the rest measurements were confirmed with independent experiments. All results were reproducible.
Randomization	Worms at desired developmental stage were randomly selected from well cultured populations.
Blinding	The investigators were not blinded. Blinding was optional but not necessary for mass spec analysis, immunoblotting analysis, lifespan assay, dauer formation assay, polyribosome profiling, and microscopy. Investigators were cautious to maintain objectivity. All experiments were conducted by at least two investigators to avoid human error.

Reporting for specific materials, systems and methods

We require information from authors about some types of materials, experimental systems and methods used in many studies. Here, indicate whether each material, system or method listed is relevant to your study. If you are not sure if a list item applies to your research, read the appropriate section before selecting a response.

Materials & experimental systems

n/a	Involved in the study
☐	☒ Antibodies
☒	☐ Eukaryotic cell lines
☒	☐ Palaeontology and archaeology
☐	☒ Animals and other organisms
☒	☐ Human research participants
☒	☐ Clinical data
☒	☐ Dual use research of concern

Methods

n/a	Involved in the study
☒	☐ ChIP-seq
☒	☐ Flow cytometry
☒	☐ MRI-based neuroimaging

Antibodies

Antibodies used	Rabbit monoclonal anti-phospho-eIF2α (Ser51) (1:1,000 dilution, clone D9G8, Cell Signaling Technology, Cat#3398S)<br>Rabbit polyclonal anti-EIF-2α (1:500 dilution, Nukazuka et al., 2008)<br>Goat anti-rabbit IgG, IRDye® 800CW Conjugated antibody (1:10,000 dilution, LI-COR Biosciences, Cat#926-32211)<br>Mouse anti-GFP (1:3,000 dilution, Roche, Cat#11814460001)<br>Mouse anti-tubulin (1:5,000 dilution, Sigma-Aldrich, Cat#T3526)<br>Goat anti-mouse IgG HRP (1:10, 000 dilution, Sigma-Aldrich, Cat#AP124)
Validation	Anti-phospho-eIF2α and anti-EIF-2α antibodies have been validated for immunoblotting in C. elegans (PMID: 18413715, 22719267, and this study).<br>Anti-GFP antibody have been validated in immunoblotting by manufacture and widely used in C. elegans (PMID: 29456082, 30353013, 26476455, 26028575).<br>Anti-tubulin antibody have been validated in immunoblotting by manufacture and used as loading control for C. elegans samples in this study.

Animals and other organisms

Policy information about studies involving animals; ARRIVE guidelines recommended for reporting animal research

Laboratory animals	C. elegans. The strains used are listed in Supplementary Table 1. All worms used were hermaphrodites. Adult day one worms were sampled in quantitative phosphoproteomics, targeted quantification by MS, immunoblotting, and polyribosome profiling. L4 - adult day one worm were visualized by microscopy. Lifespan assays followed the whole life of worms.
Wild animals	This study did not involve wild animals.
Field-collected samples	This study did not involve samples collected from the filed.
Ethics oversight	No ethical approval or guidance was required for experiments using C. elegans.

Note that full information on the approval of the study protocol must also be provided in the manuscript.
